# Opioid Receptor-Mediated Regulation of Neurotransmission in the Brain

**DOI:** 10.3389/fnmol.2022.919773

**Published:** 2022-06-15

**Authors:** Kaitlin C. Reeves, Nikhil Shah, Braulio Muñoz, Brady K. Atwood

**Affiliations:** ^1^Department of Pharmacology and Toxicology, Indiana University School of Medicine, Indianapolis, IN, United States; ^2^Department of Neuroscience, Charleston Alcohol Research Center, Medical University of South Carolina, Charleston, SC, United States; ^3^Medical Scientist Training Program, Indiana University School of Medicine, Indianapolis, IN, United States; ^4^Stark Neurosciences Research Institute, Indiana University School of Medicine, Indianapolis, IN, United States

**Keywords:** opioid, synaptic plasticity, receptor signal transduction, neurotransmission, glutamate, GABA

## Abstract

Opioids mediate their effects *via* opioid receptors: mu, delta, and kappa. At the neuronal level, opioid receptors are generally inhibitory, presynaptically reducing neurotransmitter release and postsynaptically hyperpolarizing neurons. However, opioid receptor-mediated regulation of neuronal function and synaptic transmission is not uniform in expression pattern and mechanism across the brain. The localization of receptors within specific cell types and neurocircuits determine the effects that endogenous and exogenous opioids have on brain function. In this review we will explore the similarities and differences in opioid receptor-mediated regulation of neurotransmission across different brain regions. We discuss how future studies can consider potential cell-type, regional, and neural pathway-specific effects of opioid receptors in order to better understand how opioid receptors modulate brain function.

## Introduction

Opioid drugs, which include both prescription painkillers, such as morphine and oxycodone, and illicit substances, such as heroin, are widely used and frequently misused (Kosten and George, 2002; Von Korff, 2013). An increase in prescription of opioid analgesics has precipitated an opioid crisis characterized by widespread opioid misuse, related complications, and opioid overdose (Kosten and George, 2002; Von Korff, 2013; Dahlhamer et al., 2016). This crisis presents a severe health exigency and makes salient a crucial scientific initiative to better understand the effects of opioid drugs and the mechanisms and opioid receptor systems on which these drugs act.

**FIGURE 1:**
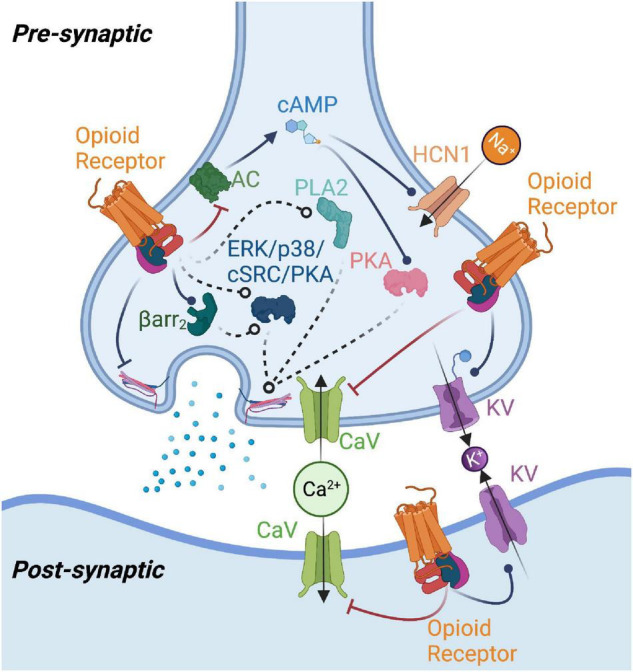
Summary of potential mechanisms of opioid receptor-mediated modulation of neurotransmission. Opioid receptor activation enhances potassium channel (KV) and inhibits calcium channel (CaV) function, reducing neurotransmitter release or producing changes in postsynaptic excitability. Opioid receptors may modulate adenylyl cyclase (AC) function to reduce cAMP levels, thereby impacting protein kinase A (PKA) and type 1 hyperpolarization-activated cyclic nucleotide-gated (HCN1) channel activity. Beta-arrestin2 (Barr2), phospholipase A2 (PLA2), as well as kinases such as p38, ERK, protein kinase C (PKC), and cSrc have been implicated in mediating opioid receptor effects on neurotransmission. Opioid receptor-mediated G protein signaling could also directly affect neurotransmitter release machinery. Figure created with BioRender.com.

Classically, opioid receptors can be categorized into one of three subtypes: mu (MOR), delta (DOR), and kappa (KOR) (Le Merrer et al., 2009). Endogenous signaling peptides activate opioid receptors: endorphins (MOR), enkephalins (primarily DOR, MOR), and dynorphins (KOR). Opioid peptides or synthetic opioid peptide derivatives are often utilized as selective opioid receptor agonists and antagonists in research. The pharmacology of these diverse ligands is reviewed elsewhere (Rasakham and Liu-Chen, 2011; Gendron et al., 2016; De Neve et al., 2021). Some commonly studied opioid receptor agonists include DAMGO (MOR), DPDPE (DOR), U69,593 or U50,488 (KOR), and the endogenous opioid peptides, met-enkephalin (MetEnk), leu-enkephalin (LeuEnk) (DOR, MOR), and dynorphin (KOR). Commonly used opioid receptor antagonists include CTAP/CTOP (MOR), naltrindole (DOR), and nor-binaltorphimine (KOR) or less selective antagonists such as naloxone. Many opioid drugs, including morphine, fentanyl, and heroin primarily activate MORs (Pasternak, 2012). Opioid receptors are Class A G protein coupled receptors (GPCRs) that couple to inhibitory G_*i/o*_ proteins (Figure 1; Stein et al., 2003; Allouche et al., 2014). These receptors transduce extracellular messages using G protein (G_α*i*_ and G_βγ_), mitogen-activated protein kinase (MAPK), and arrestin signaling pathways (Rosenbaum et al., 2009; Al-Hasani and Bruchas, 2011). Opioid receptors generally decrease neurotransmission through inhibiting voltage-gated calcium channels and activating inwardly rectifying potassium channels (Yamada et al., 1998; Al-Hasani and Bruchas, 2011). Opioid receptors can be located postsynaptically in neuronal soma and presynaptically in axon terminals (Olive et al., 1997). Postsynaptic opioid receptors inhibit neurotransmission by directly hyperpolarizing neurons, while presynaptic opioid receptors can indirectly reduce or enhance neural activity by reducing excitatory or inhibitory neurotransmission, respectively. The opioid receptors and their endogenous ligands are differentially expressed throughout the brain (Le Merrer et al., 2009; Erbs et al., 2015). Because of their widespread expression, opioid receptors are involved in a diverse array of physiological and behavioral functions, including nociception, drug reward and consumptive behavior, social memory, fear learning, stress and emotion, immune activation, and various physiological processes, such as respiration and gastrointestinal tract motility (Shippenberg et al., 1998; Drews and Zimmer, 2010; Van’t Veer and Carlezon, 2013; Leroy et al., 2017; Eisenstein, 2019; Patel et al., 2019; Toubia and Khalife, 2019; van Steenbergen et al., 2019; Robble et al., 2020; Galaj and Xi, 2021).

The expanding understanding of opioid receptor functionality, distribution, and modulation of neurotransmission has demonstrated an important role for opioids in modulating neuroplasticity. Neuroplasticity refers to the ability of the brain to change structure and function across life and in response to experience (Voss et al., 2017). The phenomenon is multi-level and can occur across networks, isolated circuits, and amongst cell populations (Citri and Malenka, 2008; Voss et al., 2017). This manifests as changes in functional and structural connectivity, the formation, migration, and elimination of neurons and glia, alterations of neuronal processes, and through synaptic plasticity (Kays et al., 2012; Kelly and Castellanos, 2014). Synaptic plasticity may be persistent with activity-dependent strengthening (long-term potentiation, LTP) and weakening (long-term depression, LTD) of connections between neurons, although there are abundant forms of short-term plasticity as well (Citri and Malenka, 2008; Atwood et al., 2014a; Motanis et al., 2018). Activity-dependent neuroplasticity is mediated by endogenous neurotransmitter systems (Viveros et al., 2007; Bliss and Cooke, 2011; Pitchers et al., 2014). Exposure to exogenous substances (e.g., neurotransmitter receptor agonists, antagonists) can also induce “chemical” plasticity (Atwood et al., 2014a). Neuroplasticity underlies many crucial processes, including learning, cognition, and neurodevelopment, and is implicated in the development of neuropathology, including mood disorders, addiction, and neurodegenerative diseases. Therefore, it is important to elucidate the role of opioid receptors in neuroplasticity (Johansson, 2004; O’Brien, 2009; Kays et al., 2012; Schaefers and Teuchert-Noodt, 2016; Voss et al., 2017). Due to their ability to modulate different neurotransmitter systems, as well as directly influencing cellular function, opioid receptors are positioned to modulate both activity-dependent plasticity and opioid drug-induced chemical plasticity (Lüscher and Malenka, 2011; Beltrán-Campos et al., 2015; Hearing et al., 2018; Hearing, 2019; Puryear et al., 2020).

The goal of this review is to demonstrate how opioid receptors modulate neurotransmission. While opioid receptors modulate a variety of neurotransmitter systems, we have limited the scope of this review to excitatory (glutamatergic) and inhibitory (often GABAergic) transmission and postsynaptic modulation of neuronal excitability. We have focused on brain regions where much work on opioid receptor-mediated regulation of neurotransmission has been performed. A summary of the literature reviewed below is provided in Table 1 and illustrated in Figure 1 as a reference for the reader. Figure 1 also illustrates how opioid receptors differentially impact neurotransmission pre- and postsynaptically. In this review, we focus on the role of opioid receptors themselves, rather than the impact of opioid drugs on general synapse and brain function. The studies reviewed herein utilized electrophysiology techniques in combination with pharmacological manipulation of opioid receptors. Studies investigating subpopulations within brain regions (i.e., input regions, cell types, projection targets) have utilized many techniques, including targeted expression of optogenetic tools, tracing strategies, and reporter animal models. We will discuss potential generalizable principles regarding opioid receptor-mediated neuroplasticity, point out broad knowledge gaps, and suggest areas of future research to advance the field, especially as it relates to cell type- and synapse-specific explorations of opioid receptor function.

**TABLE 1:** Summary of effects of mu (MOR), delta (DOR), and kappa (KOR) opioid receptor activation on neuronal excitability (postsynaptic effects), presynaptic GABA release, and presynaptic glutamate release.

	Postsynaptic effects			Presynaptic GABA			Presynaptic Glutamate		
	MOR	DOR	KOR	MOR	DOR	KOR	MOR	DOR	KOR
***Amygdala***									
**BLA**	–			+		±	±		–
**BNST**				+		+			±
**CeA**	±	±	±	+	±	+	+	±	±
**MICR**	+						±	±	–
***Brainstem/Midbrain***									
**DVM**				±			+		
**LC**	+				+				+
**MVN**	–	+	–						
**NTS**	+	–	–				±	+	+
**PAG**	±		±	+	±	±	+	–	–
**Pons**	+	–	–	+	+	+	+	–	–
**Raphe**	±		±	+	–	–	+		+
**RVM**	±	±	±	±			±		±
**SN**			+	+	+	+			+
**VTA/RMTg**	±	±	±	+	±	+	+		+
***Cortex***									
**ACC**	±	±		–	+		±	+	
**AIC**				±	±	±			
**mPFC**	+				±			±	±
**OFC**				±					
**S1**	±	±	±						
***Hippocampus***									
**CA1**	±	±	±	±	±		–	–	
**CA2**					+				
**CA3**		±	±	+	–	–	±		+
**DG**	+	+	+	+	+	+	±	–	±
***Hypothalamus***									
**AN**	±	±	±	+	±	+	+	–	+
**LH**						+			
**PO**	+		+						
**PVN**				+			+		+
**SON**	±	±	±	±		±	+		±
**VMH**	+	–	–				+	–	+
***Habenula***									
**LHb**	±			±		+	±		+
***Pallidum***									
**GP**	+	–	±	+	±	+			–
**EPN**			+			+			
**VP**	+		+	+			+		+
***Striatum***									
**DS**	–	–		±	±		±	±	+
**NAc**				±	±	±	+	±	±
***Thalamus***									
**Thalamus**	+	–	±						

*+, Identified effects of opioid receptor activation.*

*–, Identified null effect of opioid receptor activation.*

*±, Identified effects in a subpopulation of neurons or inconsistent results between studies.*

*Blanks indicate untested areas. Note that future studies may reveal heterogenous responses to opioid receptor activation where past studies have either observed widespread effects or null effects.*

## Amygdala

The amygdaloid complex is involved with emotional processing and consists of 13 nuclei, categorized as basolateral (basal, lateral, and accessory basal nuclei; BLA), cortical-like (cortical and lateral olfactory tract nuclei, periamygdaloid complex), centromedial [medial (CeM) and central nuclei (CeA)], bed nucleus of stria terminalis (BNST), or other (intercalated nuclei, anterior amygdala area, amygdalohippocampal area) (Sah et al., 2003). The amygdaloid complex has extensive connectivity across the brain, including local connectivity between amygdala nuclei (Pitkänen et al., 1997). MORs in the amygdala are involved with analgesia, fear and anxiety responses, and social behavior (Good and Westbrook, 1995; Wilson and Junor, 2008; Zhang et al., 2013; Lebow and Chen, 2016). Amygdala DORs play a role in modulating ethanol’s effects; however, a functional role of amygdala DORs may not occur until after exposure to drugs of abuse, such as ethanol and morphine (Kang-Park et al., 2007; Bie et al., 2009a,b). Amygdala KORs are involved with anxiety and fear conditioning (Knoll et al., 2011). KOR activation in the amygdala increases anxiety-like behaviors and enhances the rewarding effects of nicotine, possibly due to nicotine’s anxiolytic effect (Smith et al., 2012).

### Basolateral Amygdala

The basolateral amygdala (BLA) is the primary input region of the amygdaloid complex and receives inputs from across the brain, including hippocampus, nucleus accumbens (NAc), prefrontal cortex (PFC), thalamus, and other amygdala nuclei (Huang et al., 2021). In the lateral nucleus, MORs hyperpolarize about 50% of neurons (Sugita and North, 1993). However, a later study found MORs do not directly hyperpolarize BLA neurons, but the activity of BLA neurons is modulated by presynaptic MORs (Blaesse et al., 2015). In the lateral nucleus, MORs and DORs presynaptically inhibit GABAergic input (Sugita and North, 1993). A later study found that MOR enhances voltage-gated potassium channel (Kv) 1.2 currents and enhances action potential (AP) spike adaptation *via* G protein PLA2 signaling in lateral amygdala (Faber and Sah, 2004). MetEnk inhibits GABAergic input to the BLA from intercalated cells, presumably through MORs (Gregoriou et al., 2019). It is unknown whether MORs regulate GABA transmission from other GABAergic inputs. MOR activation reduces GABAergic input to ∼75% of CeA-projecting BLA neurons *via* activation of Kv1.1/1.2 channels. Very few CeA-projecting BLA neurons have glutamate input that is inhibited by MOR activation (Finnegan et al., 2006). On the other hand, MOR activation produces a long-lasting depression of dorsal midline thalamic glutamatergic input to BLA neurons. MOR inhibition of midline thalamic input to BLA neurons is sufficient to reduce feedforward excitation of the CeM (Goedecke et al., 2019). These studies suggest MORs may primarily modulate BLA projections to the centromedial amygdaloid nuclei; however, additional studies are needed investigating MOR modulation of BLA projections to other regions.

Kappa activation in BLA enhances presynaptic GABA transmission in a tetrodotoxin (TTX)-sensitive manner with no effect on postsynaptic responses in adolescent, but not adult rats (Przybysz et al., 2017). KORs have no effect on glutamate transmission in BLA in rats. Further exploration of the effects of KOR activation on GABA transmission in adolescent rats showed that KOR activation has a variable effect on GABA transmission with subsets of cells showing potentiation, no responses, or depression (Varlinskaya et al., 2020). Further research is needed to determine if these subsets represent sub-populations with distinct afferents/efferents. In mice, KOR activation reduces synaptic transmission from the lateral amygdala to the BLA and blocks LTP induction in the BLA (Huge et al., 2009). Overall, these studies demonstrate KORs modulate neurotransmission in the BLA and these effects demonstrate species, age, input, and output specificity.

### Bed Nucleus of the Stria Terminalis

MORs presynaptically inhibit GABAergic transmission to Ventral Tegmental Area (VTA)-projecting neurons in the ventrolateral BNST (Dumont and Williams, 2004). It is unknown whether MORs inhibit GABA transmission to non-VTA-projecting BNST neurons. MOR’s effect on glutamate transmission in the BNST is also unknown. KORs presynaptically inhibit GABAergic input from CeA to BNST *via* extracellular signal-related kinases (ERK), but not p38 (Li et al., 2012). KOR activation induces presynaptic LTD *via* p38 (not PKA or MAPK) and calcium signaling in BNST at BLA, but not PFC inputs. Despite KOR-mediated inhibition of GABA transmission, the net effect of KOR activation is to reduce AP firing of BNST neurons. This may be caused by KOR-induced inhibition of glutamate transmission in the BNST. KOR inhibits glutamate onto both dynorphin-positive and dynorphin-negative neurons but has a larger effect on dynorphin-positive neurons (Crowley et al., 2016). Overall, presynaptic MORs and KORs modulate neurotransmission in the BNST; however, while KORs inhibit both GABA and glutamate transmission, MORs have only been shown to inhibit GABA transmission.

### Centromedial Amygdala

MORs inhibit about 60% of CeA neurons, particularly those with bipolar morphology (Chieng et al., 2006). CeA neurons can be characterized as Type A or B based on the absence or presence of spike accommodation in response to prolonged depolarization current. MORs hyperpolarize a subset of Type A neurons through activation of potassium currents, whereas KORs only hyperpolarize Type B neurons (Zhu and Pan, 2004). Separate subpopulations of MOR-inhibited neurons were also inhibited by KORs or DORs. When the investigators looked at projection targets they found that MORs hyperpolarize parabrachial nucleus (PBN)-projecting neurons (Chieng et al., 2006). It is possible MOR-sensitive Type A neurons may specifically project to the CeA, although additional studies are needed to confirm this.

MORs appear to play a role in tonically inhibiting GABA release from synaptic terminals in the CeA. *In vivo* opioid exposure can also induce postsynaptic MOR-mediated inhibition of GABA current amplitudes (Kang-Park et al., 2009; Bajo et al., 2011). Specifically, periaqueductal gray (PAG)-projecting CeA neurons receive MOR-sensitive GABAergic input (Finnegan et al., 2005). Additional studies are needed to identify MOR-sensitive GABAergic inputs in the CeA. Like MORs, KOR activation inhibits GABA release in CeA in rats (Przybysz et al., 2017) and KORs may also tonically inhibit GABA release (Gilpin et al., 2014; Bloodgood et al., 2021; Khom et al., 2021). Similarly, DORs also inhibit GABA release in the CeA, but there is evidence for species differences. In one study in mice, DOR activation was shown to reduce GABA release; whereas, in another study in rats, DORs did not have an effect on GABA transmission under normal conditions, but gained the ability to do so in ethanol-treated rats (Kang-Park et al., 2007; Bie et al., 2009a). Similar to the CeA, MORs inhibit GABA transmission in the CeM; MetEnk, presumably through MORs, inhibits GABergic input from the nearby intercalated cell region of the amygdala (Gregoriou et al., 2019). MORs on the intercalated cells prevent feedforward inhibition from the BLA to the CeM (Blaesse et al., 2015). Future studies are needed to determine whether KORs or DORs inhibit GABA transmission in the CeM.

In contrast to opioid receptor-mediated effects on GABA transmission, MOR, but not DOR or KOR, activation reduces glutamate input in the CeA but not CeM (Zhu and Pan, 2005; Blaesse et al., 2015). Specifically, a small subpopulation of PAG-projecting neurons in the CeA receive MOR-sensitive glutamate input (Finnegan et al., 2005). A later study determined that MORs inhibit glutamate input to CeA neurons from the parabrachial nucleus and BLA (Kissiwaa et al., 2020), but another study found MORs do not inhibit BLA inputs to CeM neurons (Blaesse et al., 2015). Another study found MOR activation produces a transient depression of dorsal midline thalamic glutamatergic input to CeA neurons (Goedecke et al., 2019). Similar to some studies of CeA GABA transmission, DOR-mediated inhibition of glutamate release may be inducible (Bie et al., 2009a,b). A subset of BLA inputs are dually regulated by KORs and DORs, suggesting that there may be some CeA synapses that are sensitive to KORs and DORs that may not be distinguished when glutamate transmission is probed more broadly, as done previously (Zhu and Pan, 2005; Kissiwaa et al., 2020). In CeA neurons, direct parabrachial glutamatergic input to corticotropin-releasing factor (CRF) neurons is insensitive to KORs; however, KOR activation presynaptically inhibits local GABA neurons that receive parabrachial glutamatergic input, resulting in disinhibition of the CRF neurons (Hein et al., 2021).

### Medial Intercalated Cell Region

GABAergic neurons of the medial island of intercalated cells send inhibitory projections to the BLA and CeM. MORs hyperpolarize these neurons in both rats and mice (Blaesse et al., 2015; Winters et al., 2017). In rats, both MOR and DOR, but not KOR, activation can reduce glutamate release from BLA inputs to intercalated neurons. Endogenous opioid peptide release in the intercalated cell region produces presynaptic inhibition of glutamate release *via* DORs and postsynaptic hyperpolarization *via* MORs (Winters et al., 2017). On the other hand, one study found that MORs do not inhibit glutamate input from BLA to the medial intercalated cell region in mice, suggesting possible species differences (Blaesse et al., 2015). MORs also inhibit GABA transmission to intercalated neurons in rats. Direct MOR activation *via* exogenous agonist application greatly decreases local GABA transmission, although endogenous opioid peptide release has only a minor effect on this inhibitory transmission (Winters et al., 2017).

## Brainstem and Midbrain

The brainstem connects the cerebrum to the spinal cord and cerebellum. It regulates respiration, consciousness, blood pressure, heart rate, and sleep (Angeles Fernández-Gil et al., 2010). The midbrain plays key roles in sensory and motor control and has received much attention for its role in reward processing and decision making (Ruchalski and Hathout, 2012). Brainstem and midbrain express the three opioid receptors (Mansour et al., 1987; Le Merrer et al., 2009) and play major roles in drug reward, pain, and respiration (Le Merrer et al., 2009; Dahan et al., 2018; Bagley and Ingram, 2020).

### Dorsal Motor Nucleus of the Vagus

MOR activation presynaptically inhibits glutamate input, but not GABAergic input, consistent with MOR expression in terminals of glutamate, but not GABA neurons of the Dorsal Motor Nucleus of the Vagus (DVM) (Browning et al., 2002). Under normal conditions, opioid agonists fail to influence GABAergic input to these neurons; however, when cAMP signaling is engaged, MOR is trafficked to the synapse and inhibits GABA transmission. This is inhibited by disrupting cAMP and PKA signaling, suggesting that the cAMP-PKA pathway regulates trafficking of MORs into the cell surface of GABAergic nerve terminals (Browning et al., 2004). Conversely, another study found that MOR activation reduces both AP-dependent glutamate and GABA transmission in rat and mouse DVM GABA neurons. MOR activation reduces GABAergic input to DVM neurons from the nucleus of the solitary tract (NTS), potentially due to MORs on the NTS neurons (Glatzer et al., 2007). These data suggest that opioid actions may depend on the state of activation of vagal circuits.

### Locus Coeruleus

The Locus Coeruleus (LC) has a long history of studies of the impact of opioid receptor-mediated regulation of cellular function due to its high expression of MORs that inhibit LC neuron excitability (Bird and Kuhar, 1977). Recording opioid effects on ion channel function in these neurons is a common methodology for exploring opioid receptor signaling and testing hypotheses regarding receptor desensitization and opioid tolerance (for review, see Allouche et al., 2014). However, a detailed discussion of the many studies of opioid receptor desensitization and tolerance in the LC are beyond the scope of this review. In addition to MOR-mediated regulation of LC neuron excitability, KORs also function in the LC to inhibit glutamate input to LC neurons without affecting postsynaptic currents/membrane potential (McFadzean et al., 1987; Pinnock, 1992b). Local KORs within the LC are targeted by dynorphinergic neurons from other brain regions (Al-Hasani et al., 2013). LC neurons that project to the spinal cord are excited by DOR agonists *via* inhibition of presynaptic DORs on GABAergic inputs, but without an effect on glutamate input (Pan et al., 2002).

### Nucleus of the Solitary Tract

MOR, but not KOR or DOR, agonists hyperpolarize neurons in the medial, dorsomedial and dorsolateral regions of the NTS through increasing potassium conductance (Rhim et al., 1993; Glatzer et al., 2007; Poole et al., 2007). In addition to increasing potassium conductance in these neurons, MORs are able to inhibit N- and P/Q-type voltage-gated calcium channels (VGCCs) in NTS neurons (Rhim et al., 1996; Endoh, 2006). While KORs were not found to hyperpolarize neurons, KORs and MORs were found to inhibit N- and P/Q-type, but not L-type VGCCs *via* G_βγ_, but not PKA signaling (Rhim et al., 1993; Endoh, 2006). These data suggest that opioid receptors use different pathways to induce inhibition in the NTS.

MORs also inhibit synaptic transmission in the NTS. Presynaptic MORs reduce inhibitory input to NTS GABA neurons from solitary tract stimulation (Glatzer et al., 2007). Within the medial NTS, MOR activation blocks tonic GABA currents and reduces GABA release (Herman et al., 2012). Another study found that MOR-mediated local inhibition of GABA transmission was AP-dependent, suggesting MORs on cell bodies may modulate local GABA neurons (Glatzer and Smith, 2005).

Solitary tract glutamatergic input to NTS neurons is inhibited strongly by MOR and weakly by DOR and KOR agonists (Rhim et al., 1993; Glatzer and Smith, 2005; Poole et al., 2007; Boxwell et al., 2013). MOR inhibition is presynaptically localized (Glatzer and Smith, 2005). MORs equally inhibit solitary tract glutamate input to both GABAergic and non-GABAergic NTS neurons (Boxwell et al., 2013). Interestingly, MOR activation is less efficacious when GABA and glycine receptors are blocked (Boxwell et al., 2013). One study specifically recorded from NTS pro-opiomelanocortin (POMC) neurons and found that glutamate input was presynaptically regulated by MORs (Appleyard et al., 2005). On the other hand, in recordings from NTS neurons that project specifically to the PBN, DORs, but not MORs, inhibited solitary tract glutamatergic inputs (Zhu et al., 2009). One study specifically looked at tyrosine hydroxylase (TH)-positive and TH-negative neurons of the NTS (Cui et al., 2012). Like other studies they found that MORs presynaptically inhibited solitary tract input to both of these classes of neurons, but the effect was larger in TH-positive neurons. These data suggest that presynaptically expressed opioid receptors may differentially affect neurotransmitter release.

### Periaqueductal Gray

The PAG is a hot spot for opioid signaling in the brain. MORs hyperpolarize and activate G protein-couple inwardly rectifying potassium channels (GIRKs) in a subpopulation of neurons within the PAG, mostly in lateral and dorsal regions of ventrolateral PAG (vlPAG) (Chieng and Christie, 1994; Vaughan and Christie, 1997; Chiou and Huang, 1999; Vaughan et al., 2003; Chen et al., 2016). Some report that KORs have no effect on GIRK in rat PAG, while the same group report that they do in mice (Chieng and Christie, 1994; Vaughan et al., 2003), suggesting that the animal model used for studying the opioid receptor effects is important. MOR inhibits about half of lateral rostral ventromedial medulla (RVM)-projecting PAG neurons and less than a quarter of RVM-projecting vlPAG neurons through activating an outward current (Osborne et al., 1996). An investigation of the specific responses within different types of PAG neurons shows that MOR activation hyperpolarizes ventral PAG GABA neurons and reduces AP firing (Chen et al., 2016). In serotonergic (5-HT) neurons however, MOR activation hyperpolarizes the neurons but enhances AP firing. In addition to their effects on GIRKs, MORs, but not DORs or KORs, inhibit calcium channels in PAG neurons (Kim et al., 1997; Connor et al., 1999). Some CeA inputs to ventrolateral PAG are sensitive to MOR and DOR activation, responding with both excitation (20% of responses) and inhibition (25% of responses). The identities and types of responses are not clear from this study (da Costa Gomez and Behbehani, 1995). This could be due to changes in neuronal excitability described above or changes in synaptic function described below.

MORs, but not KORs or DORs, presynaptically inhibit glutamate transmission to some degree in all regions of the PAG (Vaughan and Christie, 1997; Chiou and Huang, 1999). Looking at identified cellular targets, MOR decreases glutamate input to both GABA and 5-HT neurons (Chen et al., 2016). MORs presynaptically regulate GABA in all regions of the PAG to both GABAergic and 5-HT neurons, through presynaptic activation of potassium channels and PLA2 (Vaughan and Christie, 1997; Vaughan et al., 1997; Chen et al., 2016). MORs also inhibit GABA input to ventral PAG TH-expressing neurons that project to the BNST and co-release dopamine and glutamate (Li et al., 2016). Interestingly, this involves a short-term reduction in GABA release accompanied by a more persistent inhibition of GABA transmission *via* a postsynaptic mechanism. Regarding other opioid receptors that modulate GABA transmission, there may be species differences. In rats, only MORs inhibit GABA release; whereas, in mice, KORs, but not DORs, also inhibit GABA release (Vaughan et al., 2003; Li and Kash, 2019). MORs inhibit GABA input to a greater extent than glutamate input in the PAG. The greater inhibition of GABA input overcomes MOR’s effects on glutamate input, as well as hyperpolarization, to increase AP firing of ventral PAG neurons (Chiou and Huang, 1999). The ability of DORs to inhibit GABA release in the PAG is plastic. DOR agonists have no effect on PAG GABAergic transmission in naïve mice but may be induced to do so with chronic morphine treatment (Vaughan et al., 2003; Hack et al., 2005). DOR activation may also inhibit GABA reuptake *via* GABA transporter type 1 in the PAG (Pu et al., 2012). Overall, presynaptic opioid receptors modulate neurotransmission in the PAG; however, while MORs and KORs inhibit GABA, only MORs inhibit glutamate transmission. DORs have only been shown to inhibit GABA transmission, likely using a different mechanism.

### Raphe Nuclei

There are two types of cells in the nucleus raphe magnus (NRM) that have differential responses to opioids. Primary 5-HT neurons are hyperpolarized *via* KOR-mediated GIRK activation (Pan et al., 1997; Li and Wang, 2001). Secondary GABAergic neurons are hyperpolarized by MORs also *via* GIRK activation (Pan et al., 1997; Li and Wang, 2001). MORs disinhibit primary cells through inhibiting GABA input to these KOR-sensitive cells (Pan et al., 1997). KORs also presynaptically inhibit glutamate input to both primary and secondary NRM cells (Bie and Pan, 2003).

MOR activation hyperpolarizes around 80% of non-5-HT DRN neurons and around 30% of 5-HT neurons, likely through enhancing potassium conductance. MOR activation reduces spontaneous GABA release and NMDA-induced activation of GABA release from local neurons, as well as neurons in the PAG onto 5-HT DRN neurons. As in the PAG, MORs also inhibit GABAergic input to DRN TH-expressing dopaminergic/glutamatergic neurons that project to BNST (Li et al., 2016). DOR and KOR activation have no effect on GABA transmission in these cells. One study found that MOR activation has no effect on glutamate input to 5-HT cells; whereas, a later study found that MORs are able to inhibit glutamate release and suggested this was due to experimental conditions (Pinnock, 1992a; Jolas and Aghajanian, 1997). In the positive study, they found that MORs were able to inhibit local glutamate release as well as glutamate input from the PAG (Jolas and Aghajanian, 1997). KORs are also able to inhibit glutamate input to DRN 5-HT neurons (Pinnock, 1992a). Therefore, MOR and KOR are capable of inhibiting both GABA and glutamate release, however up to the present time there is no evidence that DORs have a role in the Raphe nuclei.

### Rostral Ventromedial Medulla

In RVM there are three different cell types that show differential responses to noxious stimuli: ON cells increase firing, OFF cells decrease firing, and NEUTRAL cells show no responses (Sikandar and Dickenson, 2011). MORs and DORs inhibit ON cell responses, increase activity of OFF cells, and have no effect on NEUTRAL cells (Cheng et al., 1986; Harasawa et al., 2000). MOR activation in RVM directly inhibits ON cells. In OFF cells, there are no effects of direct MOR agonist application, suggesting that opioid-mediated excitation of OFF cells is indirect (Heinricher et al., 1992, 1994).

In measures of direct cellular responses, there are two major cell types in RVM that respond to opioids: primary cells and secondary cells. Primary cells have a wider action potential, more negative resting membrane potential, and are not inhibited by MOR agonists. Secondary cells are generally presumed to be inhibitory interneurons that serve only to regulate the activity of the output neurons, have a shorter action potential, are often firing spontaneously, and are mostly hyperpolarized by MOR agonists (Pan et al., 2000; Cleary et al., 2008). Also, primary cells are responsive to KOR activation, producing outward currents (Pan et al., 1990, 2000). Subpopulations of secondary cells are responsive to MOR activation, also producing outward currents. Almost all spinally projecting RVM neurons respond to opioids in some fashion. Subpopulations of these neurons show outward current responses to either only MOR, only KOR, or both receptor activations (Marinelli et al., 2002). Interestingly, MOR responsive secondary cells are similar to ON cells *in vivo*, and KOR responsive primary cells are similar to OFF cells (Pan et al., 1990). Non-5-HT spinally projecting neurons are almost exclusively MOR responders; whereas, 5-HT neurons have equal proportions of MOR, KOR, and MOR/KOR responders (Marinelli et al., 2002; Zhang et al., 2006; Zhang and Hammond, 2010). About two thirds of TH-expressing and TH-negative bulbospinal neurons are hyperpolarized by MOR *via* GIRK activation (Hayar and Guyenet, 1998). DORs produce outward currents in subpopulations of RVM neurons (Marinelli et al., 2005). They specifically act in a subpopulation of MOR-regulated non-5-HT spinal cord-projecting neurons, as well as subpopulations of 5-HT spinal cord-projecting neurons that have differential sensitivities to MOR and KOR activation.

MOR activation reduces GABA, but not glutamate input to primary cells (Pan et al., 1990, 2000). MOR activation reduces GABA input likely *via* inhibition of presynaptic calcium channels, but not glutamate input to RVM neurons; however, it is not clear whether these are primary or secondary neurons due to the recording conditions (Vaughan et al., 2001). Glutamatergic input to secondary cells is presynaptically inhibited by KORs (Ackley et al., 2001). MORs inhibit GABA and glutamate input to bulbospinal TH-expressing and TH-negative neurons through presynaptic mechanisms (Hayar and Guyenet, 1998). In spinal cord-projecting rat RVM neurons MORs inhibit evoked glutamate inputs in ∼50% of cells, miniature excitatory postsynaptic currents (mEPSCs) in 55% of cells, evoked inhibitory inputs in about 70% of cells, and miniature inhibitory postsynaptic currents (mIPSCs) in 100% of cells (Finnegan et al., 2004). MORs agonists frequently activate output neurons in the brain *via* disinhibition. Thus, direct inhibition of “secondary cells” disinhibits “primary cells” or output neurons, allowing them to become active (Cleary et al., 2008).

### Substantia Nigra

MORs, DORs, and KORs, have all been reported to modulate substantia nigra GABA release (Starr, 1985). KOR activation presynaptically inhibits glutamate transmission in Substantia Nigra (SN) pars reticulata (Maneuf et al., 1995). KORs can inhibit type-2 dopamine receptor (D2R)-mediated IPSCs in dopamine neurons of the SN pars compacta (Ford et al., 2007). The mechanism is unclear, given that KORs can both hyperpolarize and prevent IPSCs in the same neuron and this is not due to modulation of cAMP, kinases, calcium, or potassium channels. Overall, opioid receptors may play a role in regulating neurotransmitter release, however, more research is needed to clarify the specific actions of each of the different opioid receptors.

### Ventral Tegmental Area and Rostromedial Tegmental Nucleus

MORs hyperpolarize local GABA neurons within VTA, but not dopamine neurons, leading to greater excitation of dopamine neurons (Johnson and North, 1992). MORs can hyperpolarize secondary VTA cells, that are largely GABAergic as well as tertiary VTA cells that are NAc-projection neurons (Cameron et al., 1997). MOR-induced hyperpolarization of local GABAergic neurons rapidly desensitizes (Lowe and Bailey, 2015). In the Rostromedial Tegmental Nucleus (RMTg), also known as the tail of the VTA, neuron firing rate is reduced by MOR activation and RMTg neurons are hyperpolarized by MOR agonists, but not DOR or KOR (Lecca et al., 2011; Matsui and Williams, 2011). Contrary to other studies that find that MORs do not hyperpolarize dopamine neurons, there may be some dopamine neurons that express MORs. MORs can hyperpolarize some VTA dopamine neurons *via* increasing potassium conductance or exciting them *via* P/Q type calcium channel (Cav2.1) inhibition (Margolis et al., 2014, 2017). DPDPE-sensitive and deltorphin II-sensitive DORs are differentially expressed in different types of VTA neurons and produce a heterogeneous response: hyperpolarizing neurons *via* increasing potassium conductance or exciting neurons *via* Cav2.1, similar to MOR (Margolis et al., 2017). Interactions between the two different functional forms of DOR and MOR is not consistent between neurons, although receptor antagonist experiments reveal that functional interactions between the two different receptors do occur. KORs hyperpolarize VTA dopamine neurons *via* increasing potassium conductance (Margolis et al., 2003; Ford et al., 2007). Interestingly, only a subset of these neurons are disinhibited by MOR activation. KORs hyperpolarize VTA neurons that project to medial PFC (mPFC), but not to NAc (Margolis et al., 2006). Consistent with this, infusion of KOR agonist into VTA decreases dopamine levels in the mPFC, but not the NAc. Amygdala-projecting dopamine neurons within the VTA are also hyperpolarized by KOR activation (Margolis et al., 2008b). VTA dopaminergic neurons that project to NAc are more inhibited by KOR activation that produces outward currents (Ford et al., 2006). In contrast, VTA neurons that project to BLA (which are mostly dopaminergic) are more inhibited by MOR activation, also producing outward currents (Ford et al., 2006).

MORs reduce GABA transmission in VTA *via* inhibition of GABA release (Bergevin et al., 2002; Xiao and Ye, 2008; Matsui et al., 2014; Bull et al., 2017). MOR activation silences GABAergic VTA neuron firing and reduces evoked and spontaneous TTX-sensitive GABA release (Xiao and Ye, 2008). Knockout of MORs from NAc medium spiny neurons (MSN) reduces the ability of MORs to inhibit GABA input to local VTA GABA interneurons in VTA (Charbogne et al., 2017). Mechanisms for MOR-mediated GABA release inhibition implicate presynaptic potassium channels, beta-arrestin2, and proto-oncogene tyrosine-protein kinase Src (Bergevin et al., 2002; Bull et al., 2017). Contrary to postsynaptic MOR effects, presynaptic MORs on GABA terminals are resistant to desensitization, except when PKC is activated (Lowe and Bailey, 2015). MetEnk, presumably though MOR activation, reduces GABAergic input equally onto NAc- and BLA-projecting dopamine neurons (Ford et al., 2006). MOR regulates VTA GABAergic transmission at local interneuron synapses as well as at GABAergic inputs from the NAc, PAG, RMTg, and ventral pallidum (Matsui and Williams, 2011; Xia et al., 2011; Matsui et al., 2014; St Laurent et al., 2020). Comparing inputs to VTA dopamine neurons, one study found that MOR activation produces the greatest inhibition RMTg inputs, with very low inhibition of local interneuron input and moderate inhibition of NAc inputs (Matsui et al., 2014). A different study however concluded that MOR-modulated NAc inputs to VTA targeted VTA GABA neurons and not VTA dopamine neurons (Xia et al., 2011). MORs inhibit GABAergic input from the ventral pallidum onto both dopamine and non-dopamine neurons (Hjelmstad et al., 2013). Various forms of GABAergic plasticity occur at many of these synapses. Inhibitory LTD at RMTg-VTA dopamine neuron synapses occurs independently of MOR activation, however LTP at PAG-VTA neuron synapses is blocked by MOR activation (St Laurent et al., 2020). A variety of *in vivo* drug exposures and painful conditions shift the ability of MORs to regulate VTA GABA transmission (Shoji et al., 1999; Margolis et al., 2008a; Xiao and Ye, 2008; Guan and Ye, 2010; Madhavan et al., 2010; Graziane et al., 2013; Polter et al., 2014; Hipolito et al., 2015).

MORs and KORs non-occlusively reduce GABA input to VTA dopamine neurons (Shoji et al., 1999). GABAergic inputs from RMTg to VTA dopamine neurons are insensitive to KOR activation (Matsui and Williams, 2011). KOR activation has little effect on fast, GABA_*A*_-mediated IPSCs recorded in NAc-projecting cells, but inhibits fast, GABA_*A*_-mediated IPSCs in BLA-projecting cells (Ford et al., 2006). On the other hand, KOR activation inhibits *GABA*_*B*_-mediated slow IPSCs: KORs inhibit GABAergic input to both BLA- and NAc-projecting cells, but this effect is stronger in NAc-projecting cells.

There is a minor role for DORs in regulating VTA GABA transmission under normal conditions, but as in other brain regions, DOR-mediated inhibition of GABA transmission is inducible by *in vivo* drug exposure (Margolis et al., 2008a; Mitchell et al., 2012; Bull et al., 2017). Following stress exposure, DORs gain the ability to produce postsynaptic insertion of GABA_*A*_ receptors in a subset of neurons, *via* phosphoinositide 3-kinase (PI3K) and Akt signaling (Margolis et al., 2011). DORs do not regulate RMTg GABA synaptic inputs (Matsui and Williams, 2011).

Presynaptic MOR activation in VTA reduces glutamate transmission onto dopamine and non-dopamine neurons (Bonci and Malenka, 1999; Manzoni and Williams, 1999). In principal VTA neurons, which are primarily dopaminergic, KOR activation produces a small inhibition of glutamate input, whereas MORs produce a larger inhibition; these are non-occlusive indicating inhibition of separate populations of inputs (Margolis et al., 2005). In secondary neurons, KORs and MORs produce similar inhibition of glutamate input and the responses to each receptor activation are positively correlated. In tertiary neurons, of which a small percentage are dopaminergic, KOR and MORs similarly inhibit glutamate input, but the magnitudes of inhibition are not correlated when dually tested in each cell. These effects are largely presynaptic, although neurons with postsynaptic KOR effects are more sensitive to MOR inhibition of glutamate input and vice versa (Margolis et al., 2005). MORs also inhibit glutamate input to RMTg neurons (Lecca et al., 2011). The LTP_*GABA*_ described above can be acutely blocked by glutamatergic presynaptic MOR activation, removing the glutamate necessary for plasticity induction (Nugent et al., 2007). The role of MOR-mediated regulation of glutamate as part of the local VTA microcircuit is important to not overlook. For example, in order for morphine to activate VTA dopamine neurons, there must be a VTA glutamatergic tone for MOR-mediated inhibition of RMTg inputs to have an effect (Jalabert et al., 2011).

Altogether, these studies indicate that opioid receptor activation has a broad effect on the VTA, targeting GABA, glutamate and dopamine transmission. Therefore, VTA opioid receptors have a key clinical relevance on the control of dopamine modulation. Although there has been much investigation of opioid receptor function in VTA, there is certainly more discover regarding the cell type- and synapse-specific function of the different opioid receptors in the VTA.

## Cortex

The cortex is involved with many higher functions, including planning, processing sensory information, memory, decision making, and emotional processing (Lamotte et al., 2021; Nadeau, 2021; Kolk and Rakic, 2022). All three opioid receptors are found in the cortex; the presence, modulation of neural activity, and behavioral role of cortical opioid receptors varies across different cortical areas, and these are involved with analgesia, morphine-induced locomotor sensitization, reducing anxiety, and with the rewarding and locomotor stimulation effects of opioids (Saitoh et al., 2018; Wang et al., 2020; Jiang et al., 2021).

Many early studies of opioid receptor responses in cortex failed to identify which specific cortical regions were being explored or looked across regions non-specifically. In rat cortical brain slices MOR, DOR, and KOR agonists inhibit evoked glutamate and GABA release (Bradford et al., 1986). In addition, extracellular recordings show that MOR, DOR, and KOR agonists reduce glutamate-evoked neuronal firing (Janiri et al., 1988). However, in contrast, potassium-evoked glutamate release in rat cerebral cortex brain slices is inhibited by MOR and KOR agonists, but not DOR agonists (Nicol et al., 1996). Cultured mouse neocortical neurons express postsynaptic MORs that co-localize with AMPARs (Liao et al., 2005). Activation of these MORs inhibits glutamate transmission and induces dendritic spine retraction. Similarly, morphine inhibits glutamate release from cortical synaptosomes *via* inhibition of voltage-gated calcium channels (Yang et al., 2004). GABAergic cortical interneurons are inhibited by MORs *via* membrane hyperpolarization through increased potassium conductance (Ferezou et al., 2007). Unlike cortical GABAergic interneurons, MOR mRNA was not found in pyramidal neurons and MOR activation had no postsynaptic effects in these neurons. There was nearly a complete overlap in interneurons that responded to DAMGO and to nicotinic acetylcholine receptor (nAChR) agonist, DMPP. nAChR activation induced AP firing in interneurons and IPSCs in pyramidal neurons that were both inhibited by MOR activation. nAChR-induced GABAergic input to pyramidal cells was multiphasic, with an initial increase in IPSCs and a subsequent decrease below baseline levels. The decrease was blocked by a MOR antagonist, suggesting that nAChR activation induces enkephalin release as a form of feedback control.

### Anterior Cingulate Cortex

The Anterior Cingulate Cortex (ACC) is involved with emotion and reward processing, learning, and memory (Rolls, 2019). Met-Enk inhibits spontaneous, acetylcholine-evoked, and glutamate-evoked neuronal activity in the ACC (Palmer et al., 1978). In a subset of rat layer 5 ACC pyramidal neurons, DOR, but not MOR, activation produces direct hyperpolarization, presumably through a postsynaptic increase in potassium conductance (Tanaka and North, 1994). In comparison, MOR, but not DOR, activation hyperpolarizes a subset of non-pyramidal neurons. Met-Enk inhibits glutamate and GABA transmission in ACC neurons. This effect is mimicked by DOR, but not MOR agonist, suggesting the effect is mediated by DORs. However, a later study found MORs specifically inhibit midline thalamus inputs to layers 2/3 and layer 5 anterior cingulate cortex pyramidal neurons and parvalbumin (PV)-expressing interneurons. DORs inhibit interneurons that receive MOR-positive medial thalamic input to regulate feedforward inhibition to pyramidal neurons. Ultimately, DORs function to disinhibit thalamocortical circuits (Birdsong et al., 2019).

### Insular Cortex

The insular cortex is involved with interoception, emotion, cognition, and motivation (Namkung et al., 2017). Anterior agranular insular cortex GABAergic neurons express KORs that function to disinhibit L5 pyramidal cell inputs to the SN (Pina et al., 2020). Dynorphin decreases GABA release, but increases glutamate release, leading to disinhibition. In L5 of rat insular cortex, paired recordings between nearby GABA neurons and other GABA neurons or pyramidal cells revealed the role of MOR in regulating these synapses (Yokota et al., 2016). MOR activation reduces fast-spiking interneurons (FSI) input to other FSIs, but not to pyramidal neurons. MOR activation also reduced GABAergic input to FSIs from non-FSI neurons. In contrast, DOR activation reduced FSI input to both other FSIs and pyramidal neurons but had no effect on inhibitory transmission from non-FSI GABA neurons. All inhibition is presynaptically localized. KOR activation has no impact on FSI inputs to other insular cortex neurons.

### Medial Prefrontal Cortex

The Medial Prefrontal Cortex (mPFC) is involved with many cognitive functions and is comprised primarily of excitatory pyramidal neurons and a smaller population of inhibitory interneurons (Xu et al., 2019). MORs inhibit both non-pyramidal and pyramidal mPFC neurons, but through different mechanisms. In non-pyramidal neurons, MORs inhibit sodium conductance through a G protein, PKA, and PKC pathway (Witkowski and Szulczyk, 2006). In pyramidal neurons, MORs inhibit N-type VGCCs through a cAMP-PKA pathway (Rola et al., 2008). DORs can both inhibit and disinhibit pyramidal neuron activation. Presynaptic DORs inhibit prelimbic mPFC principal neurons through inhibiting glutamate release onto these neurons (Yamada et al., 2021). On the other hand, DORs increase GABA transmission from somatostatin-expressing interneurons to PV-expressing interneurons, which disinhibits pyramidal neurons, which MORs do not do (Jiang et al., 2021). KORs also inhibit neurotransmission in mPFC. KOR activation reduces glutamate release onto mPFC pyramidal neurons (Tejeda et al., 2013). Specifically, BLA glutamatergic inputs to mPFC are inhibited by KOR activation in *in vivo* extracellular recordings in anesthetized rodents (Tejeda et al., 2015).

### Orbitofrontal Cortex

MORs presynaptically inhibit GABA release onto pyramidal neurons of the rat ventrolateral Orbitofrontal Cortex (OFC) (Qu et al., 2015), consistent with identified expression of MOR in these GABA cells (Huo et al., 2005). MOR-LTD of presynaptic FSI PV-expressing neurons inhibit GABAergic input to pyramidal neurons of medial, but not lateral OFC. Stimulating cAMP production shifts MOR activation to produce short-term depression rather than LTD. Endogenous opioid LTD can be induced *via* moderate frequency stimulation in the presence of peptidase inhibitors, but not low frequency stimulation (Lau et al., 2020).

### Sensorimotor Cortices

MetEnk and LeuEnk inhibit a subset of sensorimotor cortical neurons, some of which are hyperpolarized by MOR agonists (Stanzione et al., 1989). In the somatosensory cortex, MORs and DORs inhibit spontaneous neuronal firing and glutamate-induced firing activity. In a subset of cells, dynorphin inhibits firing and in some recordings where dynorphin had little effect alone, it attenuated the effects of MOR and DOR activation (Janiri et al., 1988).

Overall, these studies indicate opioid receptor effects on neurotransmission and neural activity within cortical areas show great diversity across region, cell type, and neural pathways. As discussed, in some cortical regions, opioid receptor effects have been shown to occur *via* different mechanisms than in other regions. Additional studies are needed to evaluate circuit-specific opioid receptor regulation of neurotransmission throughout the cortex in order to more fully understand the impact of opioids on higher brain function.

## Hippocampus

The hippocampus is a brain region crucial to facilitating memory, learning, and spatial processing (Bird and Burgess, 2008). All three opioid receptors are heterogeneously distributed throughout the entire hippocampus and are regulated by the endogenous opioids dynorphin and enkephalin (Simmons and Chavkin, 1996).

### CA1

In measures of population spike (PS) amplitudes in CA1, both MOR and DOR enhance amplitudes in CA1 (Lee, 1978; Dunwiddie et al., 1980; French and Zieglgansberger, 1982; Valentino and Dingledine, 1982; Dingledine et al., 1983; Bostock et al., 1984; Vidal et al., 1984; Dunwiddie and Su, 1988; Neumaier et al., 1988; Moudy et al., 1989; Wimpey et al., 1989; Pieretti et al., 1994). Morphine increases hippocampal activity in CA1 in slice and in freely moving animals (Linseman and Corrigall, 1982). MORs, but not DORs or KORs, increase the duration of CA1 field potentials (Pieretti et al., 1994). The timing of MOR activation can also determine whether it can enhance CA1 function. MOR activation prevents the inhibitory effects of temporo-ammonic pathway stimulation on Schaffer collateral inputs to CA1 when the timing of stimulation of the two pathways was further apart than one theta cycle, but had no effect when timing was less than one theta cycle (McQuiston, 2011).

The effects of MOR and DOR activation are likely not due to effects on pyramidal cells themselves, although KORs might have some effects on pyramidal cell potassium currents (Madamba et al., 1999). Rather, opioid receptor-induced enhancement of population spike amplitudes is due to disinhibitory mechanisms (Zieglgansberger et al., 1979; Corrigall and Linseman, 1980; Dunwiddie et al., 1980; Neumaier et al., 1988; Lupica and Dunwiddie, 1991; Miller and Lupica, 1994; McQuiston, 2007, 2008; Tian et al., 2015). Specifically, opioids hyperpolarize GABAergic interneurons within CA1 and reduce GABA input to pyramidal neurons (Madison and Nicoll, 1988; Lupica and Dunwiddie, 1991; Lupica et al., 1992; Lupica, 1995; Capogna et al., 1996; Lafourcade and Alger, 2008; Krook-Magnuson et al., 2011; Banghart et al., 2018; Fan et al., 2019). Although DORs can inhibit GABA transmission, they do not appear to be the primary mediators of these effects (Watson and Lanthorn, 1993; Lupica, 1995). MORs can reduce feedforward and feedback inhibition, whereas DORs do not. However, both MORs and DORs are able to inhibit spontaneous GABA transmission, but not monosynaptic inhibitory postsynaptic potentials (IPSPs) (Lupica et al., 1992). However, some of the complexity may be attributable to how MORs and DORs individually regulate GABA transmission in local circuits. In CA1, MORs inhibit interneuron input to the soma, whereas DORs inhibit input to dendrites of pyramidal neurons (Svoboda et al., 1999). In support of this, one study showed that MORs inhibit FSI GABA, but not regular spiking GABA basket cell input to CA1 pyramidal neurons (Glickfeld et al., 2008; Shao et al., 2020). However, a very recent study showed that both MORs and DORs independently activate GIRK in PV neurons as well as inhibit GABA release on to pyramidal neurons (He et al., 2021). MORs hyperpolarize FSI basket cell neurons, but not regular spiking basket cell neurons. FSIs typically synapse on to somas, whereas regular spiking neurons synapse on to dendrites (Straub et al., 2016). MORs also inhibit neuropeptide Y (NPY)-expressing neurogliaform interneurons through membrane hyperpolarization (Krook-Magnuson et al., 2011). In addition, MOR specifically reduces tonic firing of the Ivy class of neurogliaform cells in CA1, reducing GABAergic input to pyramidal neurons (Krook-Magnuson et al., 2011).

The ability of MORs and DORs to disinhibit CA1 pyramidal cell function can be pathway and layer specific and may explain some of the confusing results regarding DOR activation and the broader effect of MOR activation. MORs, but not DORs, mediate feedforward inhibition from Schaffer collateral input (Rezai et al., 2013). However, DORs are expressed in interneurons within CA1 that receive input from the temporo-ammonic pathway, but not the Schaffer collateral pathway. Both MOR and DOR mediate feedforward inhibition from the temporo-ammonic pathway. While MOR enhances excitatory transmission in all layers, it is most effective at enhancing propagation through CA1 output layers (McQuiston, 2007, 2008). Stimulating CA2 pyramidal neuron input to CA1, MOR activation prevented feedforward inhibition of CA1 pyramidal neurons in deep layer and excitatory radiatum giant cells layers, but not pyramidal neurons in superficial layers through its inhibition of FSI interneurons (Nasrallah et al., 2019). MORs can also enhance excitation of pyramidal cells through enhancing excitatory responses to acetylcholine receptor activation (Kearns et al., 2001). Along with this, MOR activation can inhibit cholinergic receptor-induced cholecystokinin-expressing basket cell-mediated theta oscillations in CA1 (Nagode et al., 2014).

Opioid receptors can also have an inhibitory effect on CA1 function. LTD in CA1 is blocked by naloxone and enhanced by MOR, but not DOR or KOR activation (Francesconi et al., 1997; Wagner et al., 2001). Prior fentanyl exposure enhances LTD expression in CA1 as well (Tian et al., 2015).

### CA2

KOR and DOR activation in CA2 increases the PS following stratum radiatum stimulation (Vidal et al., 1984). Presynaptic DORs produce GABAergic LTD at FSI PV-expressing basket cell inputs to pyramidal neurons of CA2, but only short-term depression in CA1 (Piskorowski and Chevaleyre, 2013). The DOR effects enable long-lasting potentiation of CA2 transmission following high frequency stimulation of Schaffer collateral inputs that prevents the strong feedforward inhibition of CA3-CA2 transmission through DOR-mediated inhibitory LTD (iLTD) (Nasrallah et al., 2015). DOR-mediated iLTD acts as a gate for feedforward inhibition in CA2 to allow for greater activation of CA2 pyramidal neurons in response to both distal and proximal glutamatergic synaptic drive (Nasrallah et al., 2017). DOR antagonists block input timing-dependent plasticity in CA2, likely preventing the iLTD of PV-expressing inputs to pyramidal neurons (Leroy et al., 2017).

### CA3

Mossy fiber stimulation induces a potentiation of glutamate transmission in stimulated pathway of guinea pig CA3, but inhibition of nearby mossy fiber synapses (Weisskopf et al., 1993). Dynorphin presynaptically inhibits these other mossy fiber pathways; inhibiting KOR signaling allows for LTP induction in this other pathway. Dynorphin is more effective at inhibiting synapses that had undergone LTP induction than those that did not. KOR effects on CA3 LTP are mediated by a non-voltage-gated channel, calcium-dependent process (Castillo et al., 1996). KORs inhibit NMDAR-mediated currents in CA3 of guinea pig hippocampus, but DORs and MORs do not (Caudle et al., 1994). KOR modulation of mossy fiber signaling within CA3 does not occur in Sprague-Dawley rats, but does occur in other rodents. MORs equally inhibit mossy fiber transmission in rats and guinea pig (Salin et al., 1995). Species differences could be due to differential KOR expression. KOR activation enhances the voltage-dependent potassium current known as the M-current [I(M)] in rat CA3 pyramidal neurons, whereas DOR activation reduces I(M) (Moore et al., 1994). DOR antagonists inhibit IPSCs in CA3, but do not block LTP (Krug et al., 2001; Leroy et al., 2019).

MOR activation has no effect on excitatory postsynaptic potentials, but instead reduces IPSPs (Capogna et al., 1993). Activation of DORs and KORs does not inhibit IPSPs. MOR-mediated presynaptic inhibition of GABA transmission produces disinhibition that is G protein mediated and blocked by PKC activation but does not involve potassium or calcium conductance changes (Capogna et al., 1993, 1996). Later studies show that opioid analgesics that activate MORs can inhibit glutamate transmission in CA3, contrary to earlier studies (Lu et al., 2020, 2021).

### Dentate Gyrus

Morphine increases hippocampal activity in dentate gyrus in slice and in freely moving animals (Linseman and Corrigall, 1982). Within the dentate gyrus, MOR activation enhances LTP induction and naloxone prevents LTP induction of the lateral, but not medial perforant pathway (Bramham et al., 1991; Xie and Lewis, 1991; Sagratella et al., 1996; Ito et al., 2001). Interestingly, electrophysiological studies of MOR knockout mice demonstrated an inability to form LTP in the DG but not in CA1, indicating that MOR activation was crucial to LTP in the DG, but not in CA1 (Matthies et al., 2000). LTP of synaptic transmission is blocked by a DOR antagonist, without affecting potentiation of the population spike (Bramham et al., 1991; Krug et al., 2001). Perforant pathway stimulation-induced opioid peptide release with a resultant MOR- and DOR-mediated disinhibition is crucial to facilitating LTP in the dentate gyrus (Bramham and Sarvey, 1996; Ito et al., 2001). In contrast to their lack of effect in CA1, KOR activation in dentate gyrus prevents LTP induction, in contrast to MOR-induced enhancement of LTP (Sagratella et al., 1996).

MORs and DORs hyperpolarize granule cells in the dentate gyrus (Piguet and North, 1993). A study showed that activation of KORs in dentate gyrus produces hyperexcitable granule cells through a postsynaptic G protein-Kv4.2 A-type potassium current mechanism, but without a change in resting membrane potential or input resistance (McDermott and Schrader, 2011).

As in CA1–CA3 areas of hippocampus, opioid receptors in the dentate gyrus also produce disinhibition *via* their actions on GABAergic neurons; although, it appears that this disinhibition has less of an effect on LTP induction at dentate gyrus synapses. Consistent with this, MOR, DOR, and KOR activation enhance excitatory transmission in dentate gyrus granule cells, likely due to disinhibition. MOR activation is the most efficacious (Neumaier et al., 1988). MORs and DORs inhibit GABA transmission in the dentate gyrus (Piguet and North, 1993). In granule cells, MORs inhibit GABA_A_ and GABA_B_-mediated IPSCs (Shao et al., 2020). Dentate gyrus population spikes are potentiated by morphine through disinhibition, but morphine does not affect LTP induction itself (Akaishi et al., 2000).

While some studies show that KORs can enhance excitatory transmission in dentate gyrus, other studies demonstrate that KOR has more of an inhibitory effect due to effects on glutamate transmission (Neumaier et al., 1988). In guinea pig dentate gyrus, KOR activation reduces PS amplitude, while DOR and MOR had no effect. KOR activation inhibits glutamate transmission from perforant path inputs, without affecting GABA transmission (Wagner et al., 1992). A combination of brain slice electrophysiology, pharmacological probing, and anatomical lesioning revealed that KOR activation in dentate gyrus presynaptically inhibits glutamate release (Simmons et al., 1994). Activation of KORs inhibits LTP formation between the perforant path and granule cells of the guinea pig dentate gyrus (Terman et al., 1994). KORs inhibit hilar mossy fiber collateral-based LTP of guinea pig dentate gyrus granule cells, the latter of which likely occurs in a GABA_A_-dependent mechanism (Terman et al., 2000). A recent study showed MORs can inhibit glutamate transmission in dentate gyrus, specifically, NMDAR-mediated, but not AMPAR-mediated, EPSCs (Shao et al., 2020).

## Hypothalamus

The hypothalamus coordinates the neuroendocrine system (Swaab et al., 1993) and regulates metabolism, reproduction, and parental behavior (Travaglio and Ebling, 2019; Evans et al., 2021; Orikasa, 2021). Hypothalamic neurons release several neurotransmitters and peptides, including GABA, glutamate, dopamine, growth hormone-releasing hormone, gonadotropin-releasing hormone, oxytocin, and vasopressin (Kim et al., 2020). All three opioid receptors are expressed in the hypothalamus (Tavakoli-Nezhad and Arbogast, 2010; Chu Sin Chung and Kieffer, 2013).

### Arcuate Nucleus

In the Arcuate Nucleus (AN), MORs most likely inhibit only oxytocin cells, not vasopressin cells (Wakerley et al., 1983). MOR activation hyperpolarizes a subset of neurons by inducing outward current with inward rectification with no effect of TTX. Some of these MOR-sensitive cells are POMC neurons (Loose et al., 1991; Pennock and Hentges, 2011). MOR activation induces outward potassium currents in POMC neurons within the AN (Ibrahim et al., 2003). MORs act as autoreceptors, having direct effects and reducing AP firing within the recorded neuron, but can have similar effects in non-POMC neurons (Kelly et al., 1990, 1992; Lagrange et al., 1994). MORs also inhibit gonadotropin-releasing hormone-expressing neurons (Lagrange et al., 1995). DORs specifically hyperpolarize non-POMC AN neurons, while KORs do not appear to hyperpolarize AN neurons (Loose and Kelly, 1990; Pennock and Hentges, 2011). Interestingly, POMC neurons are directly inhibited by dynorphin A through activation of potassium conductance (Zhang and van den Pol, 2013; Pennock and Hentges, 2014). Previously it was considered that was due to KOR activation (Zhang and van den Pol, 2013). However, follow up studies found that this was likely due to actions of dynorphin A on MORs (Pennock and Hentges, 2014). Later studies determined KORs do hyperpolarize a subset of AN neurons, specifically NPY neurons (Zhang and van den Pol, 2013). In the AN, KOR activation reduces AP firing of neurons that express dynorphin, indicating that these receptors serve as autoreceptors (Ruka et al., 2013, 2016). Looking at synaptic transmission, in AN, MORs and KORs, but not DORs, presynaptically reduce glutamate input (Emmerson and Miller, 1999). Presynaptic MORs and KORs inhibit glutamate and GABA input to POMC neurons (Pennock and Hentges, 2011; Zhang and van den Pol, 2013). In comparison, a DOR agonist was unable to inhibit evoked GABA release but had a modest inhibitory effect on basal GABA transmission; although, it was not clear what the cause of this discrepancy was (Pennock and Hentges, 2011). MOR-mediated inhibition of GABA input is more sensitive than that of postsynaptic hyperpolarization, suggesting there may be opioid peptide concentration-dependent local circuit dynamics at play (Pennock and Hentges, 2011).

### Preoptic Hypothalamus

The preoptic hypothalamus plays a role in thermoregulation, where the neurons can be characterized by their thermosensitivity (impulses s–1°C–1) by the thermal coefficient (TC). Preoptic area neurons are hyperpolarized by MOR activation (Wagner et al., 2000). MOR activation-induced hyperpolarization reduces tonic firing activity of all types of neurons and reduces the temperature sensitivity of warm-sensitive neurons (neurons with a TC ≥ 0.8 impulses s–1°C–1) (Yakimova, 2006). In the ventrolateral preoptic area, morphine reduces the firing rate and hyperpolarizes sleep-promoting neurons (as assessed by sensitivity to norephinephrine treatment) but has no effect on non-sleep-promoting interneurons (Wang et al., 2013). The investigators found that this was due to dually activated MORs and KORs.

### Paraventricular Nucleus

In the Paraventricular Nucleus (PVN), LTD of glutamate input to vasopressin neurons is induced by paired stimulation that combines metabotropic glutamate receptor (mGluR) 1/5 activation with postsynaptic activity to cause somatodendritic dynorphin release that acts at presynaptic KORs (Iremonger et al., 2011). Presynaptic KOR activation mediates synaptic depression *via* inhibition of glutamate release downstream of calcium channel opening that the investigators predict is due to actions on release machinery (Iremonger and Bains, 2009). PVN parvocellular neurons can undergo LTD of GABAergic input *via* mGluR5-driven L-type calcium channel-dependent somatodendritic enkephalin release to act on presynaptic MORs. This iLTD requires ongoing MOR activation, as it is reversible by naloxone (Wamsteeker Cusulin et al., 2013). The released enkephalin can spread to other nearby GABA and glutamate synapses to produce pathway-independent LTD as well.

### Supraoptic Nucleus

KORs inhibit both oxytocin and vasopressin neurons of the Supraoptic Nucleus (SON), whereas MORs and DORs primarily inhibit oxytocin neurons (Inenaga et al., 1990). KORs inhibit neuron function by limiting calcium entry to reduce AP firing (Inenaga et al., 1994). In magnocellular neurons of the SON, MORS, but not KORs or DORs, inhibit postsynaptic N- and P/Q-type voltage-gated calcium channels (Soldo and Moises, 1998). In oxytocin neurons of the SON, naloxone treatment increases post spike excitability *in vivo*, suggesting an endogenous MOR tonic activation. The authors discovered that morphine treatment likely engages potassium conductances that are relieved during naloxone-precipitated opioid withdrawal, resulting in hyperexcitable oxytocin neurons, with no effects in nearby vasopressin neurons (Brown et al., 2005). MOR effects on magnocellular neurons are weak, due to inhibition of glutamate input (presynaptic), with no effects on GABA or postsynaptic effects (Liu et al., 1999). Glutamatergic and GABAergic input to magnocellular neurons is decreased presynaptically by MOR activation, with no apparent postsynaptic effects. MOR-mediated inhibition appears to be independent of inhibition of calcium channels or activation of potassium channels. KORs are also able to inhibit GABAergic input to a subpopulation of magnocellular neurons (Honda et al., 2004). Vasopressin magnocellular SON neurons were recorded in organotypic slice cultures to measure rhythmic firing patterns. KOR-mediated inhibition of glutamate release is part of the mechanism that governs the rhythmic firing of these neurons (Israel et al., 2010). This is supported by *in vivo* measures that show that KOR activation influences rhythmic firing of vasopressin, but not oxytocin, neurons of the SON (Brown et al., 1998). Dynorphin is co-released with vasopressin from the dendrites of these neurons (Brown and Bourque, 2004).

The hypothalamus is a region of great cell-type heterogeneity across hypothalamic nuclei. Both presynaptic and postsynaptic MORs and KORs have been shown to regulate hypothalamus neurons; although, the effect and mechanism varies across nuclei and cell-type. The role of DORs in the hypothalamus is less clear, as studies have found conflicting results. This may be due to a limited effect of DORs in subpopulations of hypothalamic neurons, but additional studies are needed to understand how DORs regulate neurotransmission in the hypothalamus. Most research of opioid receptor regulation of neurotransmission in the hypothalamus has focused on only a handful of hypothalamic nuclei, leaving much to be discovered. Interestingly, MORs and KORs have been shown to act as autoreceptors in multiple hypothalamic nuclei. Future studies will reveal if these opioid receptors also act as autoreceptors in other hypothalamic nuclei.

## Lateral Habenula

The lateral habenula (LHb) regulates reward, aversion, motor and cognitive function, sleep and circadian rhythms, pain, navigation, and maternal behaviors (Hu et al., 2020). It is not clear if DOR is expressed in this area, however, MORs and KORs are expressed, suggesting a role in reward, analgesic and stress responses (Gardon et al., 2014; Simmons et al., 2020). In the LHb, MOR activation has subpopulation effects: some neurons show hyperpolarization, some neurons show reduced glutamate synaptic input, and some neurons show reduced GABA input (Margolis and Fields, 2016). KOR activation in LHb presynaptically inhibits glutamate transmission, but has both inhibitory and enhancing effects on GABA transmission (Simmons et al., 2020). The net impact of KOR on regulating glutamate and GABA transmission produces KOR-mediated hyperexcitability of neurons that express hyperpolarization-activated cation currents (Ih) and decreases the excitability of Ih-negative neurons. Additional studies are needed to identify which specific LHb inputs are regulated by MORs and KORs.

## Pallidum

The pallidum is composed of the globus pallidus, entopeduncular nucleus, and ventral pallidum. Together, the pallidum has important roles in hedonic actions, motivation, and cognition (Smith et al., 2009; Saga et al., 2017). All three opioid receptor are highly expressed in the pallidum (Le Merrer et al., 2009).

### Globus Pallidus

Presynaptic MORs inhibit GABA input from dorsal striatum and from local GABAergic neurons (Stanford and Cooper, 1999). In contrast, DORs inhibit evoked local GABA transmission, but do not inhibit striatal inputs. DOR activation has no effect on AP-dependent spontaneous IPSCs, but inhibits mIPSCs. MORs, but not DORs or KORs, postsynaptically inhibit N-type VGCCs in dissociated Globus Pallidus (GP) neurons (Stefani et al., 2001). Similar to MOR, KOR activation in GP hyperpolarizes about 25% of cells and presynaptically inhibits GABAergic input from striatum and local GABAergic collaterals (Ogura and Kita, 2000). KORs have no effect on glutamate transmission, and it is unknown if DORs or KORs regulate glutamate transmission in GP.

### Entopeduncular Nucleus

A subpopulation of Entopeduncular Nucleus (EPN) neurons were hyperpolarized by dynorphin-mediated KOR activation *via* increasing potassium conductance. Electrical stimulation of the (GP) evokes GABA release from striatal and pallidal inputs to the EPN. Dynorphin equally inhibited IPSCs from both sources (short- and medium-latency IPSCs) presynaptically. Dynorphin released from striatal inputs could be an autofeedback mechanism, heterosynaptic (targeting pallidal input), or directly inhibit EPN neurons (Ogura and Kita, 2002).

### Ventral Pallidum

MORs hyperpolarize a subpopulation of Ventral Pallidum (VP) neurons, presumably through activation of potassium currents (Napier and Mitrovic, 1999). Looking at specific regional targets of VP neurons, MORs hyperpolarize GABAergic VP neurons that project to the VTA (Hjelmstad et al., 2013). *In vivo* electrophysiological recordings reveal that MOR activation reduces inhibitory GABAergic input, and excitatory substance P input from the NAc within the VP and enhances glutamate input from amygdala (Napier and Mitrovic, 1999). MOR activation produces LTD of GABA release in VP (Kupchik et al., 2014). In *in vivo* electrophysiological recordings, stimulation of VTA inputs to VP reduces firing of VP neurons. KOR and MOR activation block this, either due to direct inhibition of dopamine inputs or inhibition of non-dopaminergic VTA input (Napier and Mitrovic, 1999; Mitrovic and Napier, 2002). MORs also antagonize NAc-induced inhibitory transmission in VP (Chrobak and Napier, 1993). KORs postsynaptically inhibit GABAergic transmission from both direct pathway MSN (dMSN) and indirect pathway MSN (iMSN) inputs to VP GABA neurons. KORs generally increase GABAergic input to VP vGluT2-expressing neurons, but they could not determine if this was pre- or postsynaptically mediated and did not test specific GABAergic synaptic inputs (Inbar et al., 2020).

In summary, subpopulations of pallidal neurons are hyperpolarized by postsynaptic MORs and KORs. Presynaptic opioid receptors also modulate neural activity of pallidal neurons by inhibiting GABA release from striatal terminals and local GABAergic collaterals; although, the effect varies across opioid receptor and neurocircuit. Excitatory neurotransmission in VP is regulated by MORs and KORs, but excitatory transmission in other pallidal areas has not been shown to be modulated by opioid receptors. Most studies investigated circuit and subpopulation effects of opioid receptors in pallidum have focused on VP, therefore future studies are needed to identify specific subpopulation effects in GP and EPN.

## Striatum

The striatum is divided into dorsal and ventral regions. The dorsal striatum (DS) is heavily involved in motor control, learning, reward, and decision making (Balleine et al., 2007). The dorsal striatum is further divided into the dorsolateral (DLS) and dorsomedial striatum (DMS). The DMS is involved with goal-directed behaviors, while the DLS is involved with habitual behaviors (Lovinger, 2010; Corbit and Janak, 2016). The ventral striatum, also known as the nucleus accumbens (NAc) plays a critical role in establishing reward-associated memories to the effects of drugs and natural cues (Hyman et al., 2006). All 3 opioid receptors are highly expressed in the striatum and regulate synaptic plasticity (Le Merrer et al., 2009; Atwood et al., 2014b).

### Dorsal Striatum

Aside from an early study of opioid effects on neuronal function in dorsal striatum, there is very little indication that opioid receptors alter membrane properties of the principal dorsal striatal MSNs. One early study found that MORs slightly hyperpolarize a subset of MSNs (Jiang and North, 1992). They also found that DORs hyperpolarize a subset of non-MSN, tonically active neurons, ablating AP firing. Later studies suggest that these are likely tonically active interneurons that release acetylcholine and glutamate and their firing is inhibited by both MORs and DORs (Ponterio et al., 2013; Laurent et al., 2014). MORs reduce the firing of these cholinergic interneurons through postsynaptic G protein signaling (Ponterio et al., 2013, 2018). MOR modulation of these neurons may be circadian (Jabourian et al., 2005).

It was initially thought that opioid receptors do not inhibit GABA release in dorsal striatum(Jiang and North, 1992). However, later work found opioid receptors regulate GABA transmission in a subregion and synapse-specific manner that could be missed using more non-specific measures. MORs only inhibit GABAergic transmission within striosome subcompartments. MOR-mediated inhibition of GABA transmission within striosomes is mediated by presynaptic cAMP-PKA signaling, likely modulating presynaptic potassium channel function, and MOR inhibition is enhanced by PKC inhibition (Miura et al., 2007; Inoue et al., 2012). MORs inhibit spontaneous and TTX-insensitive GABAergic inputs in both cell types (dMSN and iMSN) (Ma et al., 2012). An elegant dissection of specific GABAergic synapses within striosomes that MORs and DORs regulate found that MORs inhibit dMSN and iMSN input to dMSNs, although inhibition of dMSN-dMSN transmission is stronger than iMSN-dMSN transmission (Banghart et al., 2015). DORs selectively inhibit iMSN input to dMSNs. Neither MOR nor DOR inhibit somatostatin-expressing interneuron input to dMSNs. DOR-mediated disinhibition of dMSNs is slightly more efficacious than MOR. MOR and DOR have little effect on GABA transmission in matrix of dorsal striatum. DOR activation produces iLTD at FSI-MSN synapses (Patton et al., 2016).

It has been known for some time that MORs and DORs inhibit glutamate release in dorsal striatum (Jiang and North, 1992). Despite MORs being enriched in striosome subcompartments of striatum, MORs equally inhibit glutamate transmission in both striosomes and matrix (Miura et al., 2007). One study that explored differences in MOR effects in dMSNs and iMSNs found that MORs reduce spontaneous glutamate release onto iMSNs in DLS, but not dMSNs (Ma et al., 2012). However, these data do not align with data from other laboratories that found more widespread MOR-mediated inhibition of glutamate release (Atwood et al., 2014b). They also reported that MORs have minimal effect on TTX-insensitive glutamate transmission in either type of MSNs in the DLS (Ma et al., 2012). MOR and DOR activation in the DLS and DMS produce antagonist-irreversible LTD in young rats and mice as well as adult mice (Atwood et al., 2014b; Fritz et al., 2018; Munoz et al., 2018, 2020). In the DLS, MOR and DOR LTD are not mutually occlusive, indicating that they inhibit different inputs. In the presence of peptidase inhibitors, electrical stimulation of glutamate release produces opioid receptor antagonist-sensitive LTD that is mGluR5 dependent. Antagonists for both MOR and DOR each partially prevent this LTD, while naloxone fully prevents this LTD. KORs may also play a role in this LTD (see below). Others have found that antidromic stimulation within the globus pallidus induces opioid peptide release (presumably enkephalins) within dorsal striatum that is sufficient to inhibit glutamate input from cortex. This was mediated by MORs, but not DORs. Paired recordings showed MSN firing could produce corticostriatal inhibition in a nearby MSN with a subpopulation showing reciprocal inhibition of cortical input (Blomeley and Bracci, 2011).

More recent work has attempted to dissect which specific glutamate synapses in the dorsal striatum are sensitive to MOR and DOR activation. In the DLS, the only cortical input that is sensitive to MOR activation are those that arise from anterior insular cortex in a mechanism that involve the activation of presynaptic HCN1 channels (Munoz et al., 2018, 2021). MORs also produce LTD in the DMS, but in this subregion the LTD is mediated by inputs from BLA, mPFC, and ACC (Munoz et al., 2020). In contrast, another recent study concluded that MORs do not inhibit ACC or mPFC inputs to DMS MSNs (Birdsong et al., 2019). The two studies were both done in mice, so it is not clear why the results are not aligned. DOR inhibits prelimbic mPFC input to DMS MSNs and motor cortex inputs to DLS MSNs (Atwood et al., 2014b; Birdsong et al., 2019). There has not been an exhaustive study of DORs effects on other cortical inputs to date. Interestingly, MORs also produce LTD of glutamate release from tonically active “cholinergic” interneurons in the DLS (Munoz et al., 2018). MORs also inhibit glutamatergic inputs from thalamus, albeit with a transient suppression rather than LTD in both DLS and DMS (Atwood et al., 2014b; Munoz et al., 2018; Birdsong et al., 2019; Reeves et al., 2021). It does not appear that DORs inhibit glutamate input from thalamus (Atwood et al., 2014b; Birdsong et al., 2019).

The early study of opioid effects on neurotransmission in dorsal striatum concluded that KORs have no effect on glutamate release (Jiang and North, 1992). However, a more recent study found that KORs can inhibit glutamate release in brain slices from young rats, specifically in dorsolateral striatum (DLS) (Atwood et al., 2014b). Activation of KORs produces an irreversible, long-lasting synaptic depression, which is similar to plasticity produced by DORs, but not MORs, in dorsal striatum. A KOR antagonist could also fully block endogenous opioid LTD in DLS, similar to the effects of naloxone, whereas MOR and DOR antagonists individually only partially blocked LTD (Atwood et al., 2014b). Given that KORs also inhibit dopamine release in dorsal striatum, it will be important to disambiguate in the future if this is due to direct activation of KORs on glutamate terminals or due to its actions on dopamine terminals which could account for the KOR antagonist on endogenous opioid LTD (Schoffelmeer et al., 1997; Szabo et al., 1999; Mamaligas et al., 2016; Hawes et al., 2017). For example, activation of Pdyn-containing dMSNs in DMS induces release of dynorphin that acts on presynaptic KORs on dopamine terminals to prevent theta burst stimulation-induced glutamatergic LTP in MSNs (Hawes et al., 2017). Similar mechanisms could account for the effects of KOR on inhibiting glutamate transmission under certain conditions.

### Ventral Striatum (Nucleus Accumbens)

The NAc can be subdivided into shell and core regions. Many studies specifically state whether measures were made in shell or core, and some provide even greater specificity. However, plenty of other studies make no distinction. Therefore, in this section where there is no specific subregion mentioned we are only able to generalize the role of opioid receptors on the specific measures discussed. There is very little evidence that opioid receptors have postsynaptic effects that influence AP firing in NAc, but much evidence that they do modulate synaptic transmission (Yuan et al., 1992; Martin et al., 1997).

As in dorsal striatum, there are some discrepant data regarding the role of MOR in regulating GABA transmission in NAc. One report demonstrates that MORs inhibit GABAergic transmission in both NAc shell and core, however, MORs have a larger effect on GABA transmission in the shell (Brundege and Williams, 2002). Another study shows that MORs inhibit GABA release in NAc shell equally in control and in forskolin-enhanced GABA release conditions (Chieng and Williams, 1998). A third study shows that MORs inhibit spontaneous GABA release similarly in D1 and D2 MSNs of the NAc core and NAc shell. Measures of TTX-insensitive GABA release show that GABA input is only inhibited in D1 MSNs in the core and D2 MSNs of the shell (Ma et al., 2012). However, a different study of the NAc shell showed that MOR activation has no effect on GABAergic input (Hoffman and Lupica, 2001). In contrast, about 50% of MSNs received input that was presumably regulated by DOR as a mixed DOR/MOR agonist was effective at blocking GABA transmission, but a MOR agonist was ineffective in these neurons. KOR activation strongly inhibits GABAergic output from D1 MSNs, but more weakly inhibits GABA output from D2 MSNs (Tejeda et al., 2017). KORs also inhibit GABA release, but with a different mechanism. KOR-mediated inhibition of GABA release is at the level of calcium entry through N-type VGCCs (Hjelmstad and Fields, 2003). Potassium channel blockade had no effect on KOR actions. Due to KOR expression on VTA dopamine neuron inputs, KORs could theoretically inhibit CIN-driven GABA co-release from VTA dopamine inputs (Britt and McGehee, 2008; Nelson et al., 2014).

MORs presynaptically inhibit glutamate release in NAc core and shell (Martin et al., 1997; Hoffman and Lupica, 2001; Brundege and Williams, 2002; Hoffman et al., 2003; James et al., 2013). Postsynaptic MOR activation was reported to enhance NMDAR, but reduce AMPAR currents (Martin et al., 1997). Regarding spontaneous glutamate release, MORs equally inhibit glutamate input to D1 MSNs in NAc core and NAc shell, but have a much larger effect on D2 MSNs of the NAc shell. MORs inhibit TTX-insensitive glutamate inputs to D1 and D2 MSNs in NAc core and NAc shell, although the effect is most robust in D1 MSNs of the shell (Ma et al., 2012). MOR’s effects on glutamate transmission in NAc may not always be neuronal in origin. MOR activation on astrocytes in NAc core induces glutamate release, producing slow inward currents *via* extrasynaptic NMDARs in nearby neurons (Corkrum et al., 2019). DOR has a minor effect on inhibiting glutamate transmission in NAc shell, perhaps only in a subset of glutamate inputs (Brundege and Williams, 2002). KORs presynaptically inhibit glutamate release on to MSNs of NAc shell without having any postsynaptic effects (Hjelmstad and Fields, 2001). KOR inhibition of glutamate transmission persist in the presence of N- and P/Q-type calcium and potassium channel blockers (Hjelmstad and Fields, 2003). Strong PFC input to NAc can produce heterosynaptic inhibition of weaker ventral hippocampal inputs, a process that is in part mediated by KORs (Brooks and O’Donnell, 2017). KOR inhibits glutamate input from BLA, but not ventral hippocampus, to D1 MSNs of the NAc shell and core, but not D2 MSNs (Tejeda et al., 2017). This effect was stronger in NAc shell than core, but was independent of D1 MSN projection target. The net effect of KOR activation at the GABA and glutamate synapses allows for KORs to decrease D1 MSN firing and disinhibit D2 MSN firing in response to BLA input. In contrast, KOR has no effect on ventral hippocampal drive of D1 MSNs, but still allows for disinhibition of D2 MSNs. The authors conclude that KOR acts as a pathway-specific filtering mechanism for BLA versus ventral hippocampal control of NAc function. KOR inhibition of glutamate transmission in NAc MSNs is lost in animals with 5 days of repeated cocaine exposure with at least up to 2 weeks of withdrawal (Mu et al., 2011). KORs also regulate glutamatergic input to PV-expressing FSIs in NAc, however, this is specific to thalamic, but not cortical inputs (Coleman et al., 2021). In addition, activation of KORs produces a postsynaptic LTD plasticity, wherein AMPARs are internalized *via* a PKA-calcineurin signaling pathway.

Altogether, these studies indicate that opioid receptor activation has little effect on membrane properties of both dorsal and ventral striatal neurons, with the exception of cholinergic interneurons. A growing body of evidence indicates that each type of opioid receptor is capable of inhibiting glutamate transmission and MORs and DORs regulate GABA transmission, although not universally at all striatal synapses. The biological relevance of synapse- and opioid receptor subtype-specific regulation of striatal excitatory and inhibitory transmission is currently unclear. Refined approaches for manipulating the expression of these receptors at specific synapses will help decipher the interplay between receptors in controlling striatal-mediated behaviors and circuit function.

## Thalamus

The thalamus acts as a relay hub for cortical sensory and motor functions, controlling perception, action and mentation (Schmitt and Halassa, 2017). MORs are highly expressed, but DORs and KORs are sparsely expressed, throughout the thalamus (Le Merrer et al., 2009; Chu Sin Chung and Kieffer, 2013; Chen et al., 2015; Bengoetxea et al., 2020).

In the thalamic reticular nucleus there are two predominant types of neurons that are both GABAergic, but display different firing properties (bursting and non-bursting). MOR activation, but not DOR or KOR, hyperpolarizes subpopulations of each class of neurons, revealing further subpopulations of neurons in this nucleus. The mechanism of hyperpolarization is due to increased potassium conductance (Brunton and Charpak, 1997). MOR activation hyperpolarizes dorsal midline thalamus neurons that project to the BLA and CeA (Goedecke et al., 2019). In the centrolateral thalamus, MOR activation, but not DOR or KOR, hyperpolarized neurons *via* increased GIRK function, independent of synaptic input. The investigators explored MOR hyperpolarization of other thalamic neurons (principal relay, midline, and intralaminar nuclei) and found widespread MOR-mediated inhibition of thalamic neurons, suggesting that the thalamus is a highly sensitive region to MOR-mediated neuronal hyperpolarization (Brunton and Charpak, 1998).

In contrast to MORs, KOR effects in the thalamus appear to be restricted to specific thalamic nuclei. KOR activation produces direct hyperpolarization of anterior paraventricular thalamic neurons through GIRKs that peak around the ages of puberty and then decrease at later ages. MOR activation hyperpolarizes these neurons; although, the effect of the MOR agonist desensitizes and produces heterologous desensitization of KOR responses (KOR responses do not desensitize independent of MOR activation) (Chen et al., 2015). Additional studies are needed to investigate potential KOR effects in other thalamic nuclei.

## Other Regions

In the above sections, we opted to review brain regions that have received the most attention. However, in our survey of the literature there are, in the context of opioid receptor-mediated regulation of neurotransmission, some other less studied brain regions or subregions that deserve further investigation and we briefly review them here.

### Lateral Hypothalamus

In the Lateral Hypothalamus (LH), local GABA neurons within the perifornical region inhibit the activity of orexin neurons. KOR activation specifically reduces this GABAergic input, as revealed by optical probing of these local GABA neurons (Ferrari et al., 2018). No studies to date have investigated MOR or DOR effects on neurons in the LH.

### Medial Vestibular Nucleus

Also known as the nucleus of Schwalbe is located in the brainstem (Highstein and Holstein, 2006). DORs, but not MORs or KORs inhibit Medial Vestibular Nucleus (MVN) neurons. DOR inhibition is *via* activation of an outward potassium current (Sulaiman and Dutia, 1998).

### Pons

Located in the brainstem, parabrachial nucleus neurons are hyperpolarized by MORs, but not DORs or KORs, likely through enhancing potassium currents (Christie and North, 1988; Cramer et al., 2021). Pontine Kölliker-Fuse nucleus neurons are hyperpolarized by MOR activation *via* GIRK activation (Levitt et al., 2015; Levitt and Williams, 2018). MORs and KORs inhibit GABA release in PBN, but only MORs regulate glutamate release (Cramer et al., 2021).

### Ventromedial Hypothalamus

Very few studies have investigated the role of opioid receptors in modulating neural function in the Ventromedial Hypothalamus (VMH) or LH. In the VMH, MORs hyperpolarize neurons to reduce cellular excitability, including those that express the leptin receptor, *via* enhancing GIRK currents (Emmerson and Miller, 1999). DORs and KORs do not appear to hyperpolarize neurons in the VMH. Presynaptic MORs strongly inhibit glutamate input to VMH neurons, whereas DORs have no effect, and KORs have only a small effect on glutamate release (Emmerson and Miller, 1999; Devidze et al., 2008). It is unknown which glutamatergic inputs to the VMH are modulated by MORs and KORs.

## General Principles, Knowledge Gaps, and Future Directions

**FIGURE 2:**
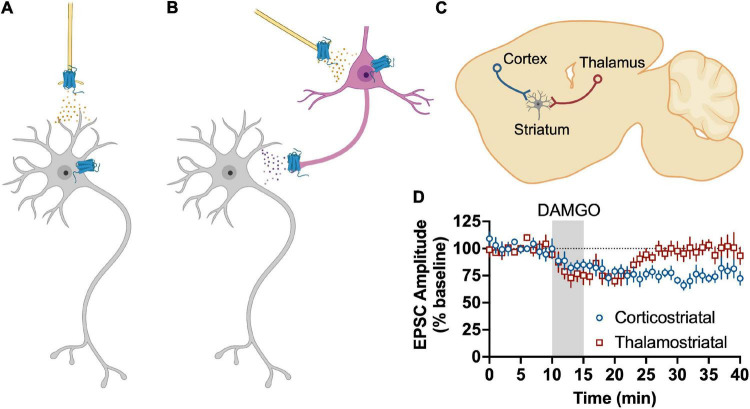
Summary of opioid receptor-mediated modulation of neurotransmission. Opioid receptor activation-mediated modulation of neurotransmission can have differential effects on neurocircuit function depending on the localization of the receptors. **(A)** Opioid receptors found on glutamatergic terminals will reduce glutamate release upon activation, thus inhibiting a postsynaptic neuron. Opioid receptors on postsynaptic neurons will generally reduce neuronal excitability. **(B)** Opioid receptors found on inhibitory neuron (e.g., GABAergic) terminals or postsynaptically will reduce inhibitory transmission, disinhibiting a postsynaptic neuron. Alternatively, opioid receptors on glutamate neurons that impinge on inhibitory neurons will reduce excitatory drive of these neurons, thus reducing inhibitory transmission and producing disinhibition through a polysynaptic mechanism. **(C)** Opioid receptors localized to different synaptic terminals can produce differential outcomes upon activation. As an example from our own work, MORs are localized to cortical and thalamic glutamatergic inputs to dorsal striatum (DS). **(D)** Upon activation by the MOR agonist, DAMGO, MORs reduce the amplitude of glutamate-mediated excitatory postsynaptic currents (EPSCs). Activation MORs on glutamate inputs from cortex produces a long-lasting reduction in EPSC amplitudes. However, activation of MORs on thalamic inputs only produces a transient reduction, despite also being a glutamatergic input to the same neurons that express the long-lasting reduction in glutamate transmission from cortical inputs. Adapted from data from Munoz et al. (2018). Figure created with BioRender.com.

Across brain regions opioid receptors play major roles in regulating glutamate and GABA release through presynaptic mechanisms and neuronal excitability through postsynaptic mechanisms. There is heterogeneity in the precise mechanisms whereby opioid receptors regulate neurotransmitter release, even within any given brain region (Figure 1). At some synapses this appears to involve inhibition of calcium channels, while at others it involves activating potassium channels. There is also evidence that diverse kinase signaling pathways may be involved at distinct synapses. These divergent mechanisms do not appear to be due to the specific identity of the opioid receptors, but rather due to the specific synaptic terminals on which the receptors are expressed. On the other hand, all three opioid receptor types appear to generally modulate neuronal excitability through their actions on potassium channels, such as GIRKs. However, local circuit effects must be considered when deciphering pre- versus postsynaptic localization of opioid receptor actions, as postsynaptic hyperpolarization can reduce local circuit neurotransmitter release (Figures 2A,B).

In order to better understand how opioid receptors modulate neurocircuit function, there is a need to identify the specific cell types that express these receptors and the subcellular localization of the receptors. Conditional knockout and fluorescent reporter transgenic mice are useful for identifying the cell types that express the various opioid receptors and how the expression of receptors within those cell types affects neurotransmission (Gaveriaux-Ruff et al., 2011; Weibel et al., 2013; Ehrich et al., 2015; Erbs et al., 2015; Chen et al., 2020). Another important consideration is identifying specific circuits that are modulated by opioid receptors. In many brain regions, opioid receptor effects on neurotransmission differ according to localization within the region, projection targets, or input regions. Optogenetic methods are increasingly accessible and are useful for identifying the specific synapses at which opioid receptors reside and how they specifically modulate neurotransmission.

It is not common for assessments of long-term opioid receptor-mediated synaptic plasticity to be performed. For many investigators, it is sufficient to determine whether a synapse is regulated by opioid receptors. However, there are missed opportunities to observe the diversity in ways in which opioid receptors modulate neurotransmission. At some synapses, activation of opioid receptors produces long-lasting effects on neurotransmission that persist even once opioid receptor antagonists are applied, which argues against persistent receptor activation. At other synapses, opioid receptor activation only produces transient responses, only lasting while the receptors are engaged (Figures 2C,D). Opioid receptors display desensitization at some synapses, while other synapses appear to be resistant to receptor desensitization. Whether a particular type of receptor in a given synapse or cell type produces long-lasting or short-term effects upon activation or desensitizes or not is a fascinating area of study that will yield rich insights into how opioids affect cognition, behavioral output, and physiological functions. Comparisons between mechanisms of synapse- and cell type-specific opioid receptor modulation of neurotransmission could also reveal novel opportunities for targeted combinatorial therapeutics. There is clearly much left to discover regarding how opioid receptors can utilize such a diverse array of mechanisms to precisely modulate neurotransmission.

## Author Contributions

All authors contributed to the conceptualization, writing, and review of this manuscript and approved it for publication.

## Conflict of Interest

The authors declare that the research was conducted in the absence of any commercial or financial relationships that could be construed as a potential conflict of interest.

## Publisher’s Note

All claims expressed in this article are solely those of the authors and do not necessarily represent those of their affiliated organizations, or those of the publisher, the editors and the reviewers. Any product that may be evaluated in this article, or claim that may be made by its manufacturer, is not guaranteed or endorsed by the publisher.
